# Beyond nerve tumors: Differential diagnoses in suspected peripheral nerve sheath tumors

**DOI:** 10.1016/j.bas.2026.105984

**Published:** 2026-02-23

**Authors:** N. Grübel, M.T. Pedro, G. Antoniadis, G. Durner, C.R. Wirtz, P. Pöschl, N. Dengler, K. Wrede, O. Gembruch, A.K. Uerschels, Peripheral Nerve Tumor Study (PNTR) Study Group

**Affiliations:** a Peripheral Nerve Unit, Department of Neurosurgery, BKH Günzburg at Ulm University, Lindenallee 2, 89312, Günzburg, Germany; b Department of Neurosurgery, University of Essen, Hufelandstraße 55, 45147, Essen, Germany; c Neurological Practice, Bruckdorferstraße 10, 93161, Sinzing, Germany; d Department of Neurosurgery, Helios Hospital Bad Saarow, Pieskower Str. 33, 15526, Bad Saarow, Germany; e Department of Neurosurgery, Johannes-Müller-Straße 7, 56068, Koblenz, Germany

**Keywords:** Peripheral nerve sheath tumor mimic, Misdiagnosis, Schwannoma, Neurofibroma, MPNST, Neuroma

## Abstract

**Introduction and research question:**

A subset of patients with suspected peripheral nerve sheath tumors (PNSTs) are ultimately found to have non-neurogenic pathologies mimicking PNSTs. This study analyzed such “drop-out” cases from the German Peripheral Nerve Tumor Registry (PNTR) to identify diagnostic pitfalls, assess imaging value, and clarify the role of intraoperative and histological findings.

**Material and methods:**

This retrospective PNTR sub-study reviewed patients initially registered with suspected PNST who were later reclassified as “drop-out” cases due to intraoperative or histopathological findings inconsistent with PNST. Patients were treated at two high-volume nerve centers (University Hospital Essen and BKH Günzburg). Clinical data, imaging, surgical notes, and histopathology were analyzed descriptively, focusing on presentation, diagnostic discrepancies, nerve involvement, and surgical strategy.

**Results:**

Of 590 registered patients, 50 (8.5%) were reclassified. The mean age was 50.8 years (range 19–90); 29 were men and 21 women. Most lesions were in the upper extremity (68%). Final diagnoses included benign soft tissue tumors (32%), malignant tumors (14%), inflammatory/immune-mediated (10%), non-neoplastic/reactive (10%), traumatic/regenerative neuromas (10%), cystic (10%), misdiagnoses/non-tumorous (10%), and vascular lesions (4%). Pain was the most frequent symptom (n = 33). Intraneural growth occurred mainly in inflammatory, reactive, and traumatic lesions, whereas non-PNSTs typically showed nerve contact only. Surgery ranged from fascicle biopsy to gross total resection.

**Discussion and conclusion:**

About one in twelve suspected PNSTs represented alternative pathologies. Red flags included absent intraneural growth, nonspecific pain, cubital tunnel clustering, and frequent biopsy need. Awareness of PNST mimics and meticulous imaging review are crucial to prevent misclassification and preserve function.

## Highlights

• One in twelve presumed PNSTs showed a non-PNST final diagnosis.
• PNST mimics commonly affected the upper extremity and ulnar nerve.
• Soft-tissue, cystic, reactive, and malignant lesions frequently mimicked PNSTs.
• High-resolution ultrasound adds key value in identifying PNST mimics.
• Targeted biopsy, not resection, is essential when nerve involvement is unclear.

## Abbreviations:

AVM: Arteriovenous Malformation
CIDP: Chronic Inflammatory Demyelinating Polyneuropathy
F: Female
GTR: Gross Total Resection
HRUS: High-resolution ultrasound
M: Male
MRI: Magnetic Resonance Imaging
MPNST: Malignant Peripheral Nerve Sheath Tumor
NF: Neurofibromatosis Spektrum Disease
LE: Lower Extremity
PR: Partial Resection
PNTR: Peripheral nerve tumor registry
PNST: Peripheral Nerve Sheath Tumor
UE: Upper Extremity

## 1 Introduction

Peripheral nerve sheath tumors (PNST)—predominantly schwannoma and neurofibroma, with malignant peripheral nerve sheath tumor (MPNST) as the aggressive counterpart—are rare, heterogeneous lesions whose imaging appearance often overlaps with non-neurogenic entities. The latter includes lesions originating from non-specific nerve tissue, such as fibrolipomas and peripheral nerve sheath ganglia. Other examples are focal inflammation with tumor-like swelling, post-traumatic or post-inflammatory neuromas, sporadic neoplasms such as xanthomas or fibrous tumors, endometriosis foci, vascular lesions, and malignant non-neurogenic neoplasms such as lymphomas and metastases (Burket and Trask, 1993), (Aru et al., 2023), (Ahlawat et al., 2014), (Prasad et al., 2016). All of these can occur intraneural or in proximity to peripheral nerves. However, this group also includes lesions that bear a considerable resemblance to peripheral nerve tumors due to specific symptoms such as radiating pain triggered by touch. Certain cutaneous tumors have been associated with significant discomfort and, in some cases, debilitating pain (Burket and Trask, 1993), (Kolbenschlag et al., 2023). Angioleiomyomas and glomus tumors are particularly important in this context (Assmus and Dombert, 2002), (Wang et al., 2022). Advanced imaging techniques, such as high-resolution ultrasound (HRUS) and Magnetic Resonance Imaging (MRI), are key to characterizing these lesions, enabling accurate diagnosis, improved patient management, and better treatment (Ahlawat et al., 2014), (Pedro et al., 2015), (Pedro et al., 2021), (Uerschels et al., 2023).

Classic MRI hallmarks (entering/exiting nerve, split-fat, target, and fascicular signs) increase confidence in a neurogenic origin. However, none are pathognomonic, and several non-PNST conditions can mimic these features (Tagliafico et al., 2019), (Kakkar et al., 2015). High-quality MRI is the cornerstone of evaluation. Contemporary guidance emphasizes standardized reporting (anatomic compartment, relationship to a named nerve, margins, internal architecture, perilesional changes) and thin-slice protocols; advanced techniques such as MR neurography and DTI can add value in selected cases (Riley et al., 2024), (Shirodkar et al., 2025), (Woertler, 2010).

Against this background, a subset of patients initially referred with a presumed PNST proved intraoperatively or histologically to harbor alternative pathologies (vascular, cystic, inflammatory/immune-mediated, benign soft-tissue, metastatic/hematologic, or reactive lesions), sometimes without accurate nerve contact. The German multicenter Peripheral Nerve Tumor Registry (PNTR) provides a framework to identify and characterize such cases (Dengler et al., 2023).

The present sub-study analyzes PNTR “drop-out” cases with preoperative suspicion of PNST but final non-PNST diagnoses, detailing imaging–clinical discrepancies, nerve relationship, and surgical strategies.

## 2 Methods

This retrospective study reviewed patients recorded from two high-volume centers of the German national PNTR (Dengler et al., 2023) who initially presented with a clinical and/or radiological suspicion of a peripheral nerve sheath tumor (PNST) and underwent surgical evaluation and treatment at the Department of Neurosurgery, University Hospital Essen, or at the Section for Peripheral Nerve Surgery, at the Department of Neurosurgery BKH Günzburg at Ulm University. Registry inclusion was triggered by referral with a suspected PNST based on external imaging and/or clinical suspicion. All patients underwent preoperative reassessment at the participating peripheral nerve centers, including neurological examination and dedicated review of cross-sectional imaging (and adjunct electrophysiology and/or HRUS when available). Only lesions that remained diagnostically ambiguous and/or clinically relevant after specialist evaluation proceeded to surgery; lesions considered unequivocally non-neurogenic were not enrolled. The analysis focused on patients who, despite an initial PNST suspicion, were reclassified as registry dropouts because intraoperative findings and/or histopathological results did not confirm a tumor diagnosis (e.g., missing nerve contact, inflammatory or vascular lesions, or other pathologies outside the scope of the registry). Data collection included reviewing clinical documentation, imaging reports, surgical protocols, and histopathological findings. Descriptive analysis focused on diagnostic discrepancies, surgical strategies, and final diagnoses. Tissue was processed according to standard neuropathological procedures of the respective centers, with additional immunohistochemical staining performed as indicated by the suspected entity; unclear cases were referred to reference neuropathology centers for confirmation. All patients provided written informed consent for inclusion in the study. The study was conducted in accordance with institutional guidelines, and ethical approval was obtained from the local ethics committees of the participating centers. The Registry is registered with the German Trials Register (www.drks.de) (Dengler et al., 2023) and got approval from the Ethics Committee of Ulm University as the seat of the registry management under the No. 249/17. Statistical analysis was not performed due to the heterogeneity of entities and small numbers per entity in several categories.

## 3 Results

The multicenter German PNTR initially enrolled up to date 590 patients with suspected peripheral nerve tumors or nerve-associated lesions. Of these, 50 patients (8.5%) were excluded due to an alternative final diagnosis or absence of nerve contact at surgery, forming the “drop-out” cohort. The remaining 540 patients constituted the PNTR population with histopathologically confirmed peripheral nerve tumors or nerve-associated lesions. This sub-study focuses exclusively on the drop-out cohort.

## 4 Baseline characteristics

The cohort comprised 29 men and 21 women, with a mean age of 50.8 years (range, 19–90). Most lesions were located in the upper extremity (n = 34), followed by the lower extremity (n = 15) and the abdomen (n = 1). Laterality showed a slight left-sided predominance (n = 28) compared to the right (n = 22). No postoperative complications were observed, such as wound-healing deficits or secondary bleeding. Baseline characteristics are summarized in Table 1.

**Table 1:** Baseline Characteristics of this PNTR Drop-Out Study Cohort.

Characteristic	Value
Peripheral nerve tumor registry, total cohort	540 total patients
Sub-study size	50/590 (8.5%)
Observation period	2016-2025
Follow-up	7.3 month
Sex (M/F)	Male: n = 29 (58%); Female: n = 21 (42%)
Age at surgery	Mean 50.8 years (range 19–90)
Lesion location	Upper extremity: n = 34 (68%); Lower extremity: n = 15 (30%); Abdominal: n = 1 (2%)
Laterality	Left: n = 28 (56%); Right: n = 22 (44%)
NF	Schwannomatosis n = 1, NF 1 n = 1
Complications	Wound healing disorder n = 0, secondary hemorrhage n = 0

NF=Neurofibromatosis Spektrum Disease, M = Male, F=Female.

Among the 50 heterogeneous lesions, eight overarching groups were defined to structure and summarize the differential diagnoses: benign soft tissue tumors (n = 16, 32%), malignant tumors (n = 7, 14%), inflammatory/immune-mediated (n = 5, 10%), non-tumorous/misdiagnoses (n = 5, 10%), traumatic/regenerative neuromas (n = 5, 10%), reactive/non-neoplastic (n = 5, 10%), cystic (n = 5, 10%), and vascular (n = 2, 24%).

## 5 Clinical characteristics by diagnostic group

Inflammatory/immune-mediated lesions (n = 5, 10%) occurred predominantly in men and were uniformly associated with sensory deficits, while motor deficits were present in three cases. Non-neoplastic/reactive lesions (n = 5, 10%) were slightly more frequent in women, with most patients experiencing pain as well as sensory and motor deficits. Benign soft tissue tumors (n = 16, 32%) represented the largest group, with a mean age of 52 years. Pain was the most common symptom in this group (14/16 cases, mostly stress-related). Surgery relieved pain in most patients, with only 4/16 experiencing residual stress-related pain, whereas neurological deficits were rare. Cystic lesions (n = 5, 10%) were primarily associated with pain and sensory deficits, while motor deficits occurred only in the two patients with ganglion cysts.

Traumatic/regenerative lesions (n = 5, 10%), all histopathologically classified as neuromas, occurred at a higher age—all patients presented with preoperative paresis, four also with sensory deficits, which persisted postoperatively. Most lesions affected major nerves, specifically the ulnar nerve (n = 3), with a predilection for the cubital tunnel, and the brachial plexus (n = 2). Vascular lesions (n = 2, 4%) were rare, occurred only in men, and were associated with sensory but not motor deficits.

**Fig. 1:**
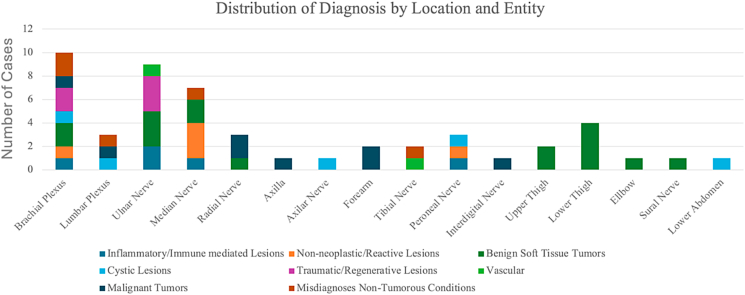
Distribution of non-PNST lesions by anatomical location and entity group. The figure highlights the broad spectrum of PNST mimics and their predilection for specific nerve locations. 1 Patient diagnosed with neuritis had two nerves (radial and ulnar nerves) involved.

Malignant tumors (n = 7, 14%) occurred at the highest mean age and showed heterogeneous pain but relatively few neurological deficits. Misdiagnosed non-tumorous conditions (n = 5, 10%) were frequently associated with preoperative pain and neurological deficits, despite the absence of histopathological confirmation, indicating underlying nerve dysfunction that was not captured histologically. These results are summarized in Table 2. Fig. 1 illustrates the anatomical distribution of the 50 “drop-out” lesions across different diagnostic groups. The ulnar nerve and brachial plexus were the most frequently affected sites, followed by the median and radial nerves. Less commonly involved locations included the lumbar plexus, tibial nerve, peroneal nerve, sural nerve, and abdominal region. Benign soft tissue tumors and traumatic/regenerative lesions accounted for most ulnar nerve cases, while brachial plexus lesions showed a wide spectrum, including malignant tumors, misdiagnoses, and inflammatory lesions. Cystic lesions were predominantly located around the tibial, peroneal, and ulnar nerves.

**Table 2:** Clinical characteristics and preoperative symptoms of patients stratified by diagnostic groups (n = 50). The table summarizes demographic data (age, sex), presence of pain at rest or under stress, sensory deficits, and motor deficits across all diagnostic categories. Mean age and age range are reported per group.

Group *(n, %)*	Age *Mean (range)*	Gender *(n)*	Pain (Stress) *(n)*	Sensoric Deficits *(n)*	Motoric Deficits *(n)*
1.Inflammatory/Immune mediated Lesions, Total *n* = 5, 10*%*	45.4 (33-61)	M, *n* = 4 F, *n* = 1	*No, n* = 0	Yes, *n* = 5	Yes, *n* = 3 No, *n* = 2
2.Non-neoplastic/Reactive Lesions, Total *n* = 5, 10*%*	42.8 (19-60)	M, *n* = 2 F, *n* = 3	Yes, *n* = 3 *No, n* = 2	Yes, *n* = 4 No, *n* = 1	Yes, *n* = 5
3. Benign Soft Tissue Tumors, Total *n* = 16, 32%	52.1 (24-77)	M, *n* = 9 F, *n* = 7	Yes, *n* = 14 *No, n* = 2	Yes, *n* = 3 *No, n* = 13	Yes, *n* = 2 *No, n* = 14
4.Cystic Lesions, Total *n* = 5, 10*%*	50.2 (35-75)	M, *n* = 2 F, *n* = 3	Yes, *n* = 4 *No, n* = 1	Yes, *n* = 3 *No, n* = 2	Yes, *n* = 2 (*only Ganglion cysts)* *No, n* = 3
5.Traumatic/Regenerative Lesions, Total *n* = 5, 10*%*	56.8 (42-90)	M, *n* = 3 F, *n* = 2	Yes, *n* = 3 *No, n* = 2	Yes, *n* = 4 *No, n* = 1	Yes, *n* = 5 *(postoperatively permanent deficits)*
6.Vascular, Total *n* = 2, 4*%*	54 (47, 61)	M, *n* = 2	Yes, *n* = 1 *No, n* = 1	Yes, *n* = 2	*No, n* = 2
7.Malignant Tumors, Total *n* = 7, 14*%*	*58.2 (42-73)*	M, *n* = 3 F, *n* = 4	Yes, *n* = 3 *No, n* = 4	Yes, *n* = 1 *No, n* = 6	Yes, *n* = 2 *No, n* = 5
8.Misdiagnoses Non-Tumorous Conditions, Total *n* = 5, 10*%*	43.2 (21-82)	M, *n* = 4 F, *n* = 2	Yes, *n* = 5	Yes, *n* = 4 *No, n* = 1	Yes, *n* = 4 *No, n* = 1
Total, *n* = 50, 100 *%*	50.8 (19–90)	M, *n* = 29 F, *n* = 21	Yes, *n* = 33 *No, n* = 17	Yes, *n* = 24 *No, n* = 26	Yes, *n* = 23 *No, n* = 27

M = Male, F=Female.

## 6 Suspected diagnosis and surgical strategy

Preoperatively, schwannoma was the most frequent working diagnosis; this included all angioleiomyomas and glomus tumors, several neuromas, and multiple vascular and malignant lesions. Nerve involvement varied by entity: intraneural growth was most common in inflammatory/immune-mediated, reactive, and traumatic/regenerative lesions, whereas benign tumors and cystic lesions were more likely to show nerve contact or none. Malignant tumors were typically extraneural, presenting with nerve contact rather than infiltration.

Surgical management differed across groups. Fascicle biopsy was performed in all inflammatory/immune-mediated and reactive lesions, while gross total resection (GTR) was most frequent in benign soft tissue tumors, cystic, vascular, and malignant lesions. Traumatic/regenerative lesions were not retrospectively added; they entered the registry because preoperative imaging and/or history led to a working diagnosis of PNST, and were reclassified as neuromas after surgery and histology. They were mainly managed with biopsy (n = 4), except for one GTR (Fig. 7). In the misdiagnosis/non-tumorous subgroup, intraoperative exploration revealed no discrete tumor, and final work-up showed either radiological misinterpretation (including one case of subclavian vein ectasia) or histopathological examination after biopsy showed nonspecific intraneural changes without evidence of inflammation or neoplasia. Accordingly, patients were managed with exploration/neurolysis (±epineurectomy/decompression) when no mass was present, or targeted epineuriotomy with fascicular biopsy when the nerve appeared pathologically altered intraoperatively. These results are summarized in Table 3.

**Table 3:** Overview of groups and final histopathological diagnoses of 50 lesions (n = 50, 100%). The table summarizes all included cases stratified by diagnostic category, with details on extremity or trunk location (upper extremity, lower extremity, trunk), preoperative diagnosis, and extent of nerve involvement (intraneural, nerve contact, or no contact). Surgical strategies are reported as gross total resection (GTR), partial resection (PR), fascicle biopsy, or neurolysis.

Groups and Final Histopathology *(n, %)* Total, *n=50, 100%*	Extremity, *(n)* (UE/LE/Trunk)	Pre-operative Diagnosis, *(n)*	Nerve Involvement^a^, *(n)* Intraneural, Nerve Contact, No Contact	Surgical strategy, *(n)*
1. Inflammatory/Immune mediated Lesions, Total *n* = 5, 10*%*				

Neuritis, *n* = 2	UE, *n* = 2	Schwannoma, *n* = 1	Intraneural, *n* = 5	Fascicle Biopsy, *n* = 5
		Perineurioma, *n* = 1		
Neuropathy, *n* = 2	UE, *n* = 1	Schwannoma, *n* = 1		
	LE, *n* = 1	Perineurioma, *n* = 1		
CIDP, *n* = 1	UE, *n* = 1	Perineurioma, *n* = 1		
2. Non-neoplastic/Reactive Lesions, Total *n* = 5, 10*%*				

Reactive perineural proliferation, *n* = 1	UE, *n* = 1	Perineurioma, *n* = 1	Intraneural, *n* = 5	Fascicle Biopsy, *n* = 5
Fibrosis, *n* = 3	UE, *n* = 3	Schwannoma, *n* = 2		
		Perineurioma, *n* = 1		
Chronic irritation, *n* = 1	LE, *n* = 1	Perineurioma, *n* = 1		
3. Benign Soft Tissue Tumors, Total *n* = 16, 32*%*				

Angioleiomyoma, *n* = 5	UE, *n* = 2	Schwannoma, *n* = 5	No Contact, *n* = 4,	GTR, *n* = 5
	LE, *n* = 3		Nerve Contact, *n* = 1	
Angiolipoma, *n* = 2	UE, *n* = 1	Schwannoma, *n* = 2	Nerve Contact, *n* = 1,	GTR, *n* = 2
	LE, *n* = 1		No Contact, *n* = 1	
Leiomyoma, *n* = 1	UE, *n* = 1	Schwannoma, *n* = 1	No Contact, *n* = 1	GTR, *n* = 1
Fibrous Dysplasia, *n* = 1	UE, *n* = 1	PNST, *n* = 1	Nerve Contact, *n* = 1	Tumor Biopsy, *n* = 1
Fibroblastic Tumor, *n* = 1	UE, *n* = 1	Neurofibroma, *n* = 1	Intraneural, *n* = 1	GTR, *n* = 1
Glomus Tumor, *n* = 4	UE, *n* = 2	Schwannoma, *n* = 4	No contact, *n* = 4	GTR, *n* = 4
	LE, *n* = 2			
Mature Cell Lipoma, *n* = 1	UE, *n* = 1	MPNST, *n* = 1	Nerve Contact, *n* = 1	GTR, *n* = 1
Plexiform Xanthoma, *n* = 1	UE, *n* = 1	Schwannoma, *n* = 1	Intraneural, *n* = 1	GTR, *n* = 1

4. Cystic Lesions, Total *n* = 5, 10*%*				

Ganglion Cyst, *n* = 2	UE, *n* = 2	PNST, *n* = 2,	Intraneural, *n* = 1	GTR, *n* = 2
			Nerve Contact, *n* = 1	
Endometriosis Cyst, *n* = 2	Trunk, *n* = 1	Schwannoma, *n* = 1	No Contact, *n* = 1	GTR, *n* = 1
	LE, *n* = 1	MPNST, *n* = 1	Nerve contact, *n* = 1	PR, *n* = 1
Epidermoid Cyst, *n* = 1	LE, *n* = 1	Schwannoma, *n* = 1	Nerve contact, *n* = 1	GTR, *n* = 1

5. Traumatic/Regenerative Lesions, Total *n* = 5, 10*%*				

Neuroma, *n* = 5	UE, *n* = 5	Schwannoma, *n* = 4	Intraneural, *n* = 5	GTR, *n* = 1
		Perineurioma, *n* = 1		Biopsy, *n* = 4
6. Vascular, Total *n* = 2, 4*%*				

AVM, *n* = 1	LE, *n* = 1	Schwannoma, *n* = 1	Intraneural, *n* = 1	PR, *n* = 1
Thrombosed Aneurysm, *n* = 1	UE, *n* = 1	Schwannoma, *n* = 1	Nerve contact, *n* = 1	GTR, *n* = 1

7. Malignant Tumors, Total *n* = 7, 14*%*				

Chordoma, *n* = 1	LE, *n* = 1	PNST, *n* = 1	Nerve contact, *n* = 1	PR, *n* = 1
Melanoma Metastasis, *n* = 1	UE, *n* = 1	MPNST, *n* = 1	No Contact, *n* = 1	Biopsy, *n* = 1
Thyroid Carcinoma Metastasis, *n* = 1	UE, *n* = 1	Schwannoma, *n* = 1	Nerve contact, *n* = 1	GTR, *n* = 1
Lymphoma, *n* = 2	UE, *n* = 2	Schwannoma, *n* = 2	Nerve Contact, *n* = 2	GTR, *n* = 2
Giant Cell Tumor, *n* = 1	UE, *n* = 1	Schwannoma, *n* = 1	Nerve contact, *n* = 1	GTR, *n* = 1
Synovial Sarcoma, *n* = 1	UE, *n* = 1	Schwannoma, *n* = 1	Nerve contact, *n* = 1	GTR, *n* = 1

8. Misdiagnoses Non-Tumorous Conditions, Total *n* = 5, 10*%*				

Unspecific Changes, *n* = 1	UE, *n* = 1	Perineurioma, *n* = 1	Intraneural, *n* = 1	Fascicle Biopsy, *n* = 1
No Tumor, radiologic misinterpretation, *n* = 4	UE, *n* = 2	PNST, *n* = 3	Intraneural, *n* = 4	Fascicle Biopsy, *n* = 2
	LE, *n* = 2	Perineurioma, *n* = 1		Neurolysis, *n* = 2

a Intraoperative nerve contact (None/Contact/Intraneural), GTR = Gross Total resection, AVM = arteriovenous malformation, PR=Partial Resection, PNST=Peripheral Nerve Sheath Tumor, MPNST = Malignant Peripheral Nerve Sheath Tumor.

## 7 Discussion and case presentations

This sub-analysis of the German PNTR emphasizes the diagnostic heterogeneity of lesions initially classified as peripheral nerve sheath tumors (PNSTs) but excluded after surgery and histopathological work-up. At 8.5%, the “drop-out” cohort is a relevant subgroup demonstrating clinically and radiologically relevant pitfalls in preoperative PNSTs assessment. The cases, from our cohort, represent rare yet clinically meaningful PNST mimics; integrating MRI with intraoperative findings provides practical reference for clinicians and radiologists facing diagnostically challenging lesions. Because such entities are rarely reported, their systematic documentation helps refine differential diagnosis, identify potential pitfalls, and support safer, function-preserving treatment strategies.

## 8 Spectrum of mimicking lesions

Benign soft tissue tumors represented the largest subgroup (32%), followed by malignant tumors (14%) and a range of reactive, inflammatory, cystic conditions, or vascular lesions underscoring that both neoplastic and non-neoplastic conditions can clinically and radiologically resemble PNSTs.

MRI remains the gold-standard diagnostic tool, which is why characteristic features such as the split-fat sign and target sign may provide valuable diagnostic clues (Shirodkar et al., 2025), (Yao et al., 2023). The strongest indicator of a neurogenic tumor remains continuity with a nerve or location along a typical nerve distribution (Riley et al., 2024), (Shirodkar et al., 2025). In our cohort, most lesions appeared adjacent to nerves on imaging but showed no intraoperative nerve contact, underscoring the need for thin-slice, high-resolution studies. Misinterpretation of imaging or clinical findings can delay diagnosis; any mismatch between history, examination, and the typical profile of PNSTs should prompt reassessment.

**Fig. 2:**
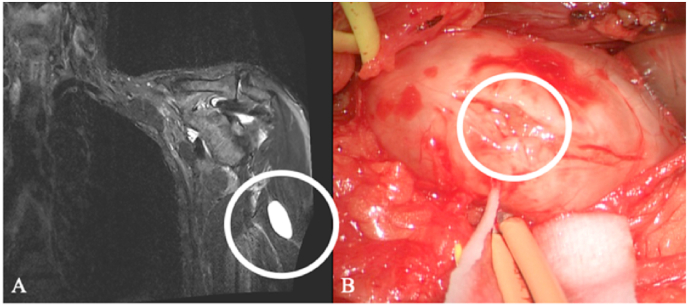
Ganglion cyst in a 64-year-old man, initially suspected to be an axillary-nerve schwannoma. (A) Coronal Fat-suppressed T2 MRI shows a well-circumscribed, hyperintense ovoid mass in the lateral upper arm (white circle). (B) Intraoperatively, it proved to be a ganglion cyst with typical gelatinous fluid and was completely excised, with histology confirming the diagnosis. The patient had preoperative pain at rest and sensory deficits, which fully resolved by 3 months with no residual lesion.

Ganglion cysts, the most common tumor-like lesions, are increasingly recognized correctly due to more widespread nerve imaging (Savić et al., 2025). Ganglion cysts at their typical sites (such as the fibular head with peroneal nerve involvement) (Knoll et al., 2019) are often correctly diagnosed preoperatively; however, it is equally essential to consider rare localizations (Fig. 2).

**Fig. 3:**
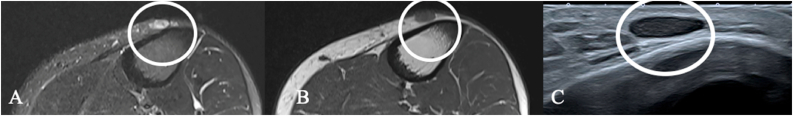
Glomus tumor. 48-year-old, pretibial mass. (A–B) MRI shows a small, well-circumscribed superficial nodule in the pretibial subcutaneous soft tissues (circles), T2-hypointense and vividly enhancing (A). (C) Ultrasound demonstrates a lens-shaped hypoechoic lesion without direct nerve contact. Schwannoma was initially suspected; intraoperatively and histologically, the diagnosis was glomus tumor.

Glomus tumors are rare, benign tumors derived from the glomus body and often diagnosed late (Assmus and Dombert, 2002), (Biswas et al., 2024). They typically arise in highly vascular skin regions (e.g. subungual area or the deep dermis of the palms, wrists, forearms, and feet), occur in about 5% of neurofibromatosis type 1 (NF1) as NF1-related tumors and are clinically suggested by the triad of pain, focal tenderness, and cold hypersensitivity, as with many other benign nerve sheath tumors (Assmus and Dombert, 2002), (Wang et al., 2022), (Biswas et al., 2024), (Mensing and Mensing, 2001). In our cohort of 4 patients with this infrequent entity, ultrasound revealed an almost pathognomonic appearance: a lens-shaped, hypoechoic subcutaneous lesion (Fig. 3) that was accessible for surgical removal.

**Fig. 4:**
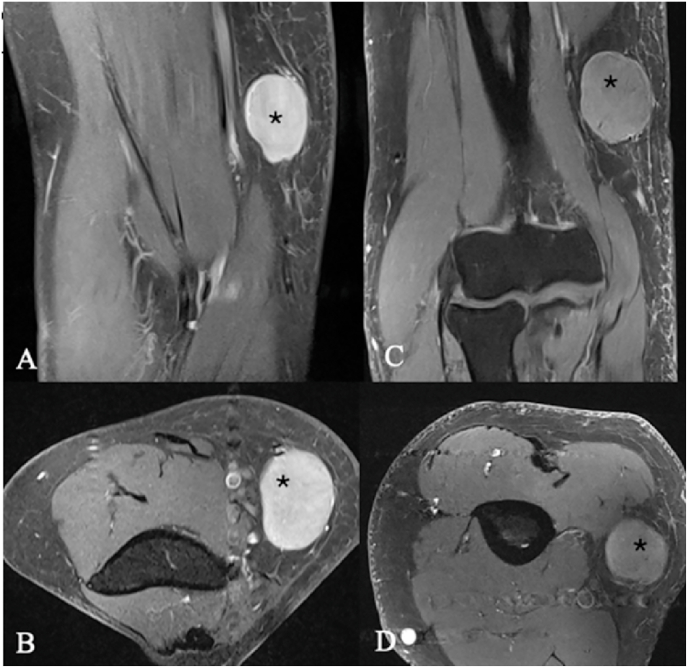
Lymphoma. MRI of two patients with soft-tissue tumors along the course of the medial antebrachial cutaneous nerve. (A–B) Patient 1, female, 73 years old (longitudinal and axial planes). (C–D) Patient 2, male, 55 years old (longitudinal and axial planes). In both cases, a schwannoma of the medial antebrachial cutaneous nerve was initially suspected on imaging. Intraoperatively, the lesions were found to be closely associated with the nerve but not arising from it; each tumor circumferentially encased the nerve. Gross-total resection was achieved in both patients, and histopathology confirmed lymphoma. The asterisk (∗) marks the tumor.

**Fig. 5:**
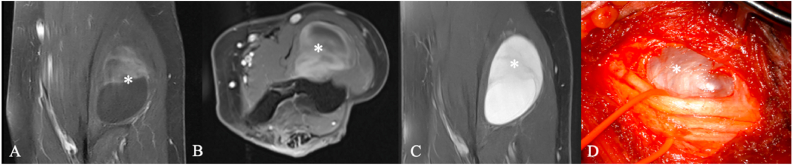
Synovial sarcoma. A 54-year-old woman with a slowly enlarging mass in the left cubital fossa over three years. MRI suggested a radial nerve schwannoma (A–C, ∗marks the tumorous lesion). Intraoperatively, the lesion appeared partly cystic and partly solid, well demarcated, and displaced but did not infiltrate the radial nerve (D, ∗). Histopathology revealed a synovial sarcoma.

Furthermore, various systemic diseases and malignancies like metastatic solid tumors, leukemia, or lymphoma (Fig. 4) can also involve peripheral nerves (Grübel et al., 2024a). Fig. 5 presents the case of an unusual synovial sarcoma in the left cubital fossa.

**Fig. 6:**
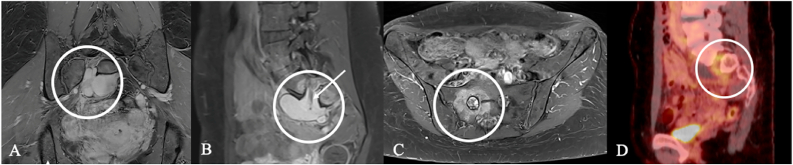
Endometriosis cyst of an 41-year-old woman with severe, partly cycle-dependent lumbosacral pain. (A, B) Pelvic MRI show a circumscribed, multiloculated cystic lesion along the lumbosacral plexus and S1 root (circles) with adjacent sacral bone (C). (D) FDG-PET demonstrated focal uptake along the S1 root (mean SUV 3,6) raising suspicion for MPNST and prompting planning of extended biopsy. Intraoperatively, old hemorrhagic fluid was encountered; histology confirmed an endometriotic cyst rather than a nerve-sheath tumor. The long clinical course and absence of motor and sensory deficits also argued against malignancy.

Endometriosis (Fig. 6) can affect the lumbosacral plexus or sciatic nerve, with symptoms often cyclical (Uppal et al., 2017), (Moeser et al., 1990).

**Fig. 7:**
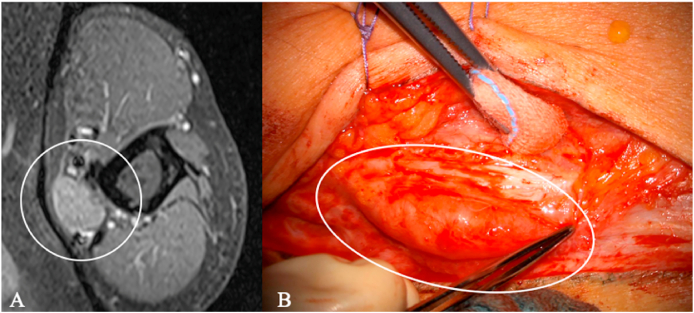
Ulnar nerve neuroma in a 52-year-old man. (A) Axial contrast-enhanced MRI at the elbow shows a well-demarcated intraneural mass along the ulnar nerve (circle). (B) Intraoperative view demonstrates a fusiform enlargement of the ulnar nerve (ellipse). A schwannoma was initially suspected based on imaging and the history of a previously resected, more proximal lesion of the same nerve. Microsurgical complete resection was performed.

Post-traumatic lesions such as end-bulb neuromas and neuromas in continuity (Fig. 7) are typically identifiable on cross-sectional imaging. They show T2 hyperintensity and variable T1 hyperintensity compared with muscle, with strong post-contrast enhancement.

**Fig. 8:**
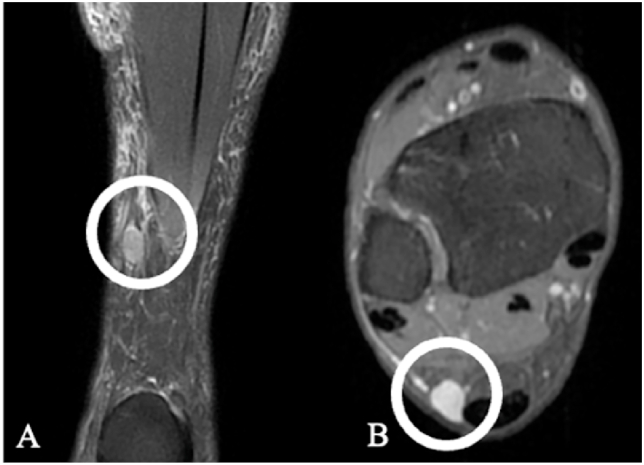
Angioleiomyoma. Female, 53 years. MRI shows a small, well-circumscribed subcutaneous mass (white circle) along the sural nerve: (A) longitudinal alignment with the nerve; (B) axial eccentricity next to the neurovascular bundle. Schwannoma was suspected. Intraoperatively, the lesion was highly vascularized and completely resected; histology confirmed angioleiomyoma.

**Fig. 9:**
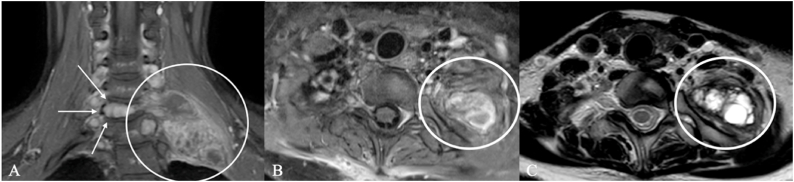
Fibrous dysplasia in a 24-year-old woman. MRI shows a T1-hypointense, avidly enhancing lesion centered at the C7 vertebra with cortical destruction/osteolysis and extension toward the left brachial plexus. (A) Coronal post-contrast T1-weighted image depicts the enhancing mass and osseous involvement (arrows). (B) Axial post-contrast T1-weighted image confirms the enhancing lesion along the left brachial plexus. (C) Axial T2-weighted image demonstrates multilocular cystic components. White circles delineate the tumor. Intraoperatively, calcifications were noted and an open biopsy was performed; histopathology confirmed fibrous dysplasia, adjuvant pamidronate therapy was started, and aside from pain there were no pre- or postoperative sensory or motor deficits.

Small superficial tumors, including the ‘LEND AN EGG’ group (leiomyoma, eccrine spiradenoma, neuroma, dermatofibroma, angiolipoma, neurilemmoma, endometrioma, glomus tumor, and granular cell tumor), may clinically and radiologically mimic PNSTs (Burket and Trask, 1993), (Aru et al., 2023), among them, cutaneous leiomyomas are rare smooth muscle tumors with angioleiomyoma (Fig. 8) being the most common subtype arising from the vascular tuna media (Lee et al., 2020). Sarcoidosis rarely infiltrates nerves, but up to 1% of patients may develop granulomatous neuropathy (Tavee, 2022). Rare mimics include fibrous dysplasia (Fig. 9), xanthomas, fibroblastic/myofibroblastic tumors, and chordomas, which are typically sacrococcygeal or skull base tumors, and can rarely involve peripheral nerves (Hatem, 2014), Fig. 10.

**Fig. 10:**
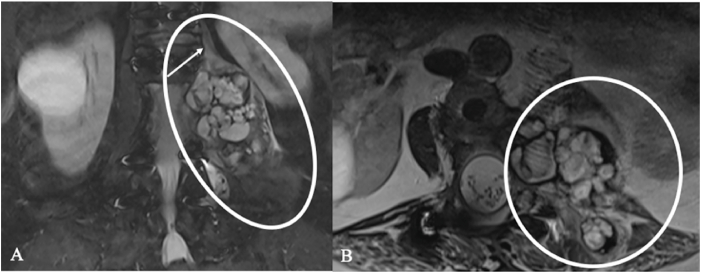
Chordoma in a 72-year-old woman. MRI demonstrates a multilobulated lesion with predominantly high signal on fluid-sensitive sequences and internal septations along the lumbosacral plexus. (A) The coronal image shows the cluster-like mass tracking cranio-caudally (white ellipse; arrow indicates the plexus course). (B) Axial image depicts the conglomerate abutting on the plexus elements (white ellipse). The imaging appearance initially raised suspicion for a peripheral nerve sheath tumor. A partial resection was performed. Sensory and motor deficits were already present preoperatively.

**Fig. 11:**
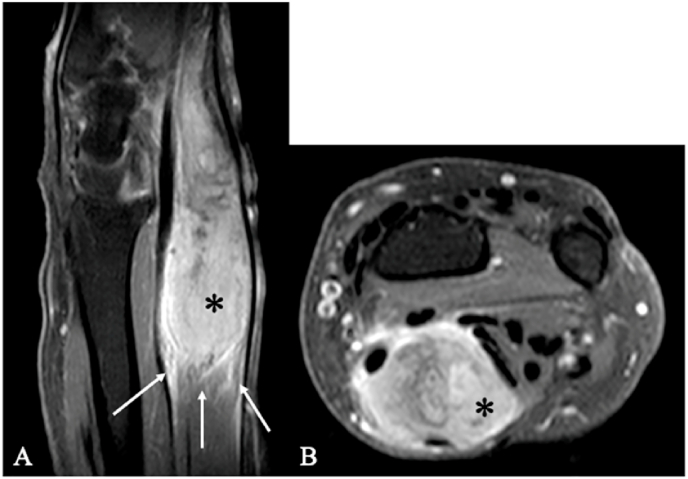
Plexiform xanthoma of the right wrist. (A) Longitudinal contrast-enhancing MRI of a 59-year-old woman shows a multilobulated lesion along the volar wrist with infiltration of the surrounding soft tissues (B). Axial image shows the lesion occupying the carpal tunnel (asterisk). A schwannoma of the median nerve was initially suspected on imaging. Intraoperatively, the tumor infiltrated the median nerve with contiguous spread into adjacent muscle. A complete resection and reconstruction were performed using a sural nerve interposition graft, resulting in postoperative new paresis and sensory deficits.

Tendinous and subcutaneous xanthomas are associated with hypercholesterolemia, typically occur in familial hyperlipidemia with accelerated atherosclerosis and most commonly involve the Achilles tendon, followed by the extensor tendons of the hand and elbow (Song et al., 2015). Fig. 11 demonstrates the MRI of a plexiform xanthoma of the right wrist.

**Fig. 12:**
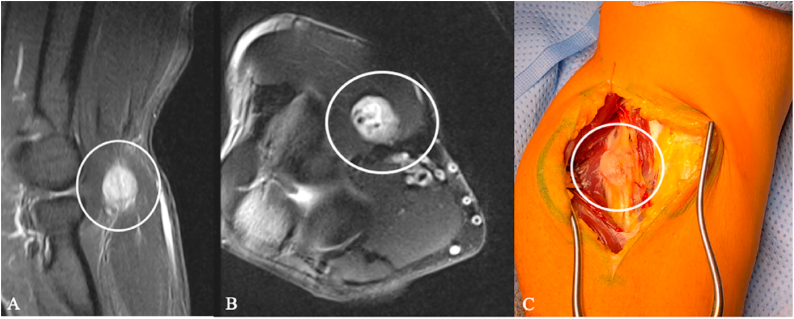
Fibroblastic soft-tissue tumor of the radial nerve. (A, B) MRI and intraoperative findings in a 66-year-old man showed a circumscribed but inhomogeneous mass (circle) along the right radial nerve at the elbow, initially suspected to be a neurofibroma (C) intraoperatively the lesion was infiltrative and fascicle-engulfing, requiring complete resection with sural-nerve interposition graft reconstruction, and the patient had marked preoperative finger extensor weakness with motor and sensory deficits present both before and after surgery.

Fibroblastic and myofibroblastic lesions (Fig. 12) are rare soft-tissue tumors that can occur at any age but are more common in children, and their true incidence may be higher than assumed. They range from benign entities (e.g. fibrous hamartoma of infancy) to locally aggressive tumors (e.g. desmoid fibromatosis) rarely metastasizing sarcomas (dermatofibrosarcoma protuberans, myxoinflammatory fibroblastic sarcoma, and low-grade myofibroblastic sarcoma); and malignant forms (e.g. sclerosing epithelioid fibrosarcoma and low-grade fibromyxoid sarcoma) (Manoharan et al., 2015).

## 9 Clinical and surgical implications

Pain was the most frequent presenting symptom across entities; benign soft tissue tumors and cystic lesions often caused stress-related pain that improved after surgery, whereas traumatic neuromas are associated with persistent pain and sensory symptoms, ongoing neurological deficits, and limited functional recovery even after excision (Kolbenschlag et al., 2023), (Remy et al., 2024). The primary goal of surgery is preservation of function. Although postoperative deficits may occur even after resection of benign nerve tumors, these risks should be minimized by meticulous microsurgical technique, fascicle-sparing dissection when feasible, and a low threshold to limit resection and perform targeted biopsy when intraoperative findings are atypical.

Malignant tumors in the drop-out cohort were characterized by relatively few neurological deficits, which can be explained by their predominantly extraneural growth pattern with nerve contact rather than infiltration, whereas malignant tumors arising from peripheral nerves are often associated with severe deficits (Guha et al., 2018).

## 10 Role of biopsy in inflammatory and reactive lesions

Inflammatory and reactive changes were exclusively intraneural and therefore required fascicular biopsy, underscoring the need for structured surgical decision-making because these interventions carry functional risks without offering definitive therapeutic benefit through resection. On MRI, both Perineurioma and CIDP may present as long-segment nerve enlargement with contrast enhancement; but clinically, perineurioma usually causes slowly progressive paresis over years and only rarely sensory deficits (Uerschels et al., 2020), (Brand et al., 2021). Electrodiagnostic studies play a significant role in the preoperative evaluation since CIDP leads to typical abnormalities of a demyelinating lesion, which usually cannot be found with primary nerve tumors (Van Den Bergh et al., 2021).

## 11 Diagnostic pitfalls and anatomical predilection

**Fig. 13:**
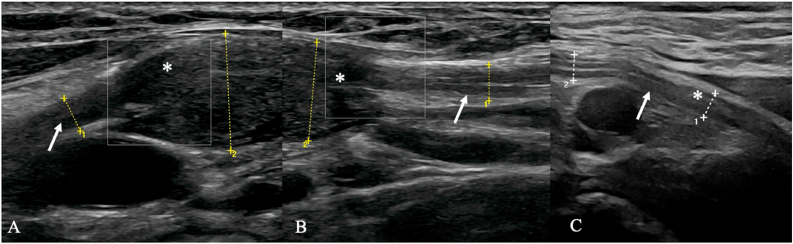
High-resolution ultrasound of a 58-year-old woman with a lesion arising from the medial fascicle of the median nerve, clinically presenting with hypesthesia of digits 3–4 (ulnar aspects), thenar atrophy, and paresis of the flexor pollicis longus, while pronator teres, flexor carpi radialis, and sensation in digits 1–2 remained intact. Initial diagnosis was a suspected schwannoma. (A–B) Preoperative longitudinal ultrasound images showing the intraneural lesion (∗). (C) Postoperative follow-up demonstrated a spontaneous regression on ultrasound. Histopathology revealed fibrotic tissue.

Heterogeneous, reactive, and inflammatory lesions carry an inherent risk of sampling error because of their focal, segmental pathology, which can affect histological interpretation, especially when malignancy is suspected (Prasad et al., 2016), (Van Den Bergh et al., 2021), (Szymanski et al., 2021), (Graham et al., 2019). Many entities contain only small, diagnostically relevant foci, so focal fascicular biopsies can sample non-representative tissue, resulting in misclassification or inconclusive histology. Small or poorly targeted biopsy may therefore miss the full disease spectrum. To reduce diagnostic uncertainty in suspected PNSTs, a comprehensive preoperative work-up is essential: electrophysiology helps differentiate demyelinating from axonal disorders, and HRUS can assess nerve continuity, intraneural versus extra neural localization, and nerve contact (Fig. 13). Access to HRUS is not widely available and is operator dependent; it excels for superficial lesions but is limited for deep or retroperitoneal tumors. Combined with MRI, HRUS substantially improves the accuracy of preoperative classification.

Lesions most often involved the ulnar nerve and brachial plexus, areas prone to PNSTs and their mimics. Prasad et al. highlighted the value of precise anatomical localization for intraneural vascular anomalies (Prasad et al., 2016) and proposed an imaging classification distinguishing subparaneurial from subepineurial lesions, with implications for resectability and recurrence risk. Although vascular mimics are rarely reported (Grübel et al., 2024b), (Rodrigues et al., 2024), Fig. 14 illustrates a thrombosed ulnar artery aneurysm resembling an ulnar nerve schwannoma on MRI.

**Fig. 14:**
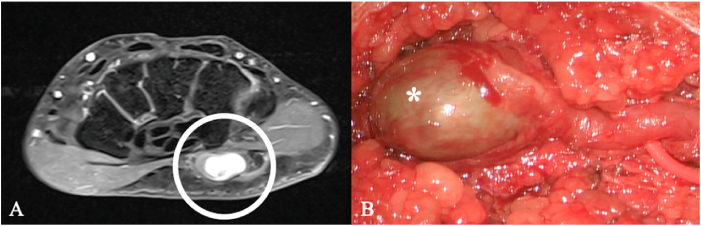
Thrombosed Aneurysm in a 47-year-old man, initially suspected to be an ulnar-nerve schwannoma: (A) Axial fat-suppressed T2-weighted MRI shows a well-circumscribed subcutaneous mass at the ulnar neurovascular bundle (white circle), but (B) intraoperatively it proved to be a thrombosed ulnar artery aneurysm (∗), which was completely excised with preservation of both nerve and vessel.

## 12 Possible indicators of PNST mimics

Key indicators against PNST in this cohort were lack of intraneural localization, nonspecific symptoms—most commonly pain—and a high biopsy rate. Clustering at the cubital tunnel (ulnar nerve) was an additional red flag. In such cases, differential diagnoses should include painful cutaneous tumors (e.g., glomus tumor, leiomyoma, endometriosis, granular cell tumor, or angiolipoma), and complete preoperative imaging should be carefully reviewed (Burket and Trask, 1993), (Mensing and Mensing, 2001).

Patients with ambiguous imaging findings, atypical symptoms, or unclear electrophysiology should be referred to specialized peripheral nerve physicians, where multidisciplinary evaluation—including neurology, radiology, neurosurgery, and neuropathology—to reduce misdiagnosis and unnecessary surgery (Guedes et al., 2021), (Guedes et al., 2023).

## 13 Limitations and strengths

Limitations: This retrospective sub-study includes small case numbers per entity, limiting statistical power, subgroup analyses, and generalizability. Sampling error is an additional limitation, particularly in small intraneural lesions and when malignancy is suspected. Due to the marked heterogeneity of diagnosis detailed histopathological and immunohistochemical descriptions eace case could not be provided. Neurological deficits were recorded only as present/absent, without standardized severity grading (e.g., MRC), precluding analyses of deficit extent (including benign vs. malignant comparisons), and rehabilitation/physiotherapy was not systematically recorded, limiting interpretation of functional outcomes.

Strengths: The cohort captures extremely rare nerve- and non-nerve-associated lesions—some with no prior case reports—identified from an extensive registry of >500 PNTR patients, allowing targeted selection of the most relevant differential diagnoses for peripheral nerve–related masses.

## 14 Conclusion

Awareness of these differential diagnoses can be highly valuable for physicians and pathologists. Reliable high-resolution imaging, neurophysiological assessment, and close collaboration among radiology, neurology, and surgeons are crucial. If no definite tumor is seen intraoperatively, biopsy rather than resection should be favored. Red flags include the absence of intraneural growth, nonspecific pain patterns, frequent biopsy requirements, clustering at the cubital tunnel, lack of nerve contact on imaging, and subcutaneous localization, in which painful cutaneous tumors should be considered. Implementing these considerations may reduce unnecessary resections and optimize functional outcomes.

## Contribution

Grübel N: Conception and design of the study, acquisition of data, writing and drafting the article, Pedro MT: acquisition of data, drafting the article. Antoniadis G: acquisition of data, Durner G: acquisition and interpretation of data, Wirtz C: analysis and interpretation of data, Pöschl P: acquisition of data, writing the article, Dengler N: acquisition of data, Wrede K: analysis and interpretation of data, Gembruch O: analysis and interpretation of data, Uerschels AK: acquisition of data, writing and drafting the article.

## Declaration of generative AI and AI-assisted technologies in the manuscript preparation process

During the preparation of this work, the authors used ChatGPT (OpenAI, San Francisco, CA, USA) in order to assist with language editing and manuscript refinement. After using this tool/service, the authors reviewed and edited the content as needed and take full responsibility for the content of the published article.

## Declaration of competing interest

The authors declare that they have no known competing financial interests or personal relationships that could have appeared to influence the work reported in this paper.
